# Application of Plant-Growth-Promoting Fungi *Trichoderma longibrachiatum* T6 Enhances Tolerance of Wheat to Salt Stress through Improvement of Antioxidative Defense System and Gene Expression

**DOI:** 10.3389/fpls.2016.01405

**Published:** 2016-09-15

**Authors:** Shuwu Zhang, Yantai Gan, Bingliang Xu

**Affiliations:** ^1^College of Grassland Science, Gansu Agricultural UniversityLanzhou, China; ^2^Key Laboratory of Grassland Ecosystems, The Ministry of Education of ChinaLanzhou, China; ^3^Sino-U.S. Centers for Grazingland Ecosystems SustainabilityLanzhou, China; ^4^Gansu Provincial Key Laboratory of Aridland Crop Sciences, Gansu Agricultural UniversityLanzhou, China

**Keywords:** *Trichoderma longibrachiatum* T6, wheat seedling, salt stress, plant-growth-promoting, antioxidative defense system and gene expression

## Abstract

Soil salinity is a serious problem worldwide that reduces agricultural productivity. *Trichoderma longibrachiatum* T6 (T6) has been shown to promote wheat growth and induce plant resistance to parasitic nematodes, but whether the plant-growth-promoting fungi T6 can enhance plant tolerance to salt stress is unknown. Here, we determined the effect of plant-growth-promoting fungi T6 on wheat seedlings’ growth and development under salt stress, and investigated the role of T6 in inducing the resistance to NaCl stress at physiological, biochemical, and molecular levels. Wheat seedlings were inoculated with the strain of T6 and then compared with non-inoculated controls. Shoot height, root length, and shoot and root weights were measured on 15 days old wheat seedlings grown either under 150 mM NaCl or in a controlled setting without any NaCl. A number of colonies were re-isolated from the roots of wheat seedlings under salt stress. The relative water content in the leaves and roots, chlorophyll content, and root activity were significantly increased, and the accumulation of proline content in leaves was markedly accelerated with the plant growth parameters, but the content of leaf malondialdehyde under saline condition was significantly decreased. The antioxidant enzymes-superoxide dismutase (SOD), peroxidase (POD), and catalase (CAT) in wheat seedlings were increased by 29, 39, and 19%, respectively, with the application of the strain of T6 under salt stress; the relative expression of SOD, POD, and CAT genes in these wheat seedlings were significantly up-regulated. Our results indicated that the strain of T6 ameliorated the adverse effects significantly, protecting the seedlings from salt stress during their growth period. The possible mechanisms by which T6 suppresses the negative effect of NaCl stress on wheat seedling growth may be due to the improvement of the antioxidative defense system and gene expression in the stressed wheat plants.

## Introduction

Salt stress is one of the major abiotic stresses that affects plant growth, development, and crop yield (Ma et al., 2012; Rivero et al., 2014). Wheat (*Triticum aestivum*), the most important cereal crop in the world, is considered to be salt sensitive (Tian et al., 2015). Grown under salt conditions, wheat plants often produce a significantly low grain yield with poor quality. Studies have shown that salt stress can induce several morphological, physiological, and metabolic responses of plants, which causes ROS stress and osmotic stress in plants, leading to increased peroxidation of lipid and antioxidant enzyme inactivation (Garg and Manchanda, 2009). Also, plants grown under salt stress conditions usually synthesize several kinds of soluble compounds including soluble sugars and proteins, which may help adjust osmoticum, retain cell turgor, and stabilize cell structures (Bartels and Sunkar, 2005).

At the present time, about 6% of the arable land on the earth is salt affected, especially in arid and semiarid regions (Bui, 2013). This seriously threatens global agricultural sustainability and food security. Thus, it is critically important to develop effective and practical techniques to alleviate the negative effects of salt stress on plant growth and development. Conventional breeding and transgenic technology have been used to develop new cultivars with improved salt tolerant traits, but breeding salt tolerance has not been successful (Phang et al., 2008). The long breeding cycle and low breeding efficiency for the quantitative trait presents challenges. Transgenic technology has the ability to incorporate salt tolerant genes in new plant materials (Sairam and Tyagi, 2004; Sahi et al., 2006; Lu et al., 2007), but the effectiveness has been low and also enveloped in controversy (Zou et al., 2015). Furthermore, gene loss, high cost, and other regulatory issues are the main bottlenecks for commercial transgenic plants use (Glick, 2007). A newer attempt is to apply exogenous compounds to decrease the negative effect of abiotic stress; this technique has been shown to increase plant tolerance to salt stress, such as using oligochitosan (Ma et al., 2012), nitric oxide and calcium nitrate (Tian et al., 2015), chitooligosaccharides (Zou et al., 2015), and jasmonic acid (Qiu et al., 2014) in wheat, as well as gibberllic acid and calcium chloride in linseed (*Linum usitatissimum*; Khan et al., 2010), and ascorbic acid in broad bean (*Vicia faba*; Younis et al., 2010). These exogenous compounds have been shown to improve the salt tolerance of plants, but the exact physiological mechanisms are unknown. A new, innovative technique that has attracted a great deal of attention in recent years, is to use plant-growth-promoting bacteria and fungi to induce plant resistance to abiotic stress. It is an effective approach for enhancing plant tolerance to salt stress and this approach may play a role in the development of sustainable agricultural systems. *Trichoderma* spp. is one of the important groups of rhizosphere microorganisms, which can impart some beneficial effects on promoting plant growth and development (Harman et al., 2004; Qi and Zhao, 2013). The *Trichoderma* species have also been known to be used by plants as biological control agents for controlling different species of plant fungus diseases for decades (Harman et al., 2004). Mastouri et al. (2010) have reported that *Trichoderma afroharzianum* T22 can enhance tomato (*Solanum lycopersicum*) seed germination under biotic and abiotic stresses, alleviating oxidative damage in osmotic stressed seedlings. However, the underlying mechanisms responsible for the alleviation of oxidative damage remain to be explored. Little information is available regarding the potential and possible mechanisms of plant-growth-promoting fungi T6 in enhancing the tolerance of wheat to salt stress.

Our previous studies show that *Trichoderma longibrachiatum* has a higher potential of parasitic and lethal effects against *Heterodera avenae* (Zhang et al., 2014b), but its effects on wheat are fairly high in promoting plant growth and nematode control (Zhang et al., 2014a). However, the previous studies failed to determine the possible mechanism of T6 enhancing the tolerance of wheat to salt stress. Therefore, the objectives of the present study were to (i) evaluate the effect of the strain of T6 on wheat growth under various levels of salt stress, and (ii) explore the possible mechanism of T6 in response to salt stress at physiological, biochemical, and molecular levels.

## Materials and Methods

Experiments were carried out at the Pratacultural Engineering Laboratory of Gansu Province. The replicated experiment was firstly conducted in 2014, and in order to obtain solid results, the same and entire experiment was repeated for a second run in 2015.

### Fungal Inoculum Preparation

The salt tolerance strain of T6 was obtained from the Laboratory of Plant Pathology, Gansu Agricultural University. The conidia suspension of T6 was prepared according to the method of Zhang et al. (2014b). Final suspension of 1.0 × 10^8^ conidia per ml were prepared and stored at 4°C.

### Plant Material and Treatment Conditions

All the experiments were conducted with wheat (cv. Yongliang 4). Consistent sizes of wheat seeds were surface-sterilized with a 1% NaOCl solution for 10 min, and then thoroughly washed with distilled water six times over. Wheat seeds were soaked in T6 or sterilized distilled water overnight for 12 h, and then transferred to Petri dishes with two layers of moist gauze for germination at 25°C for 24 h in the dark. Fifty germinated wheat seeds were planted in each transparent box (12 by 12 by 5 cm) which was filled with water agar containing 0 and 150 mM NaCl (control and NaCl, respectively) and were grown in an incubator at a day/night cycle of 16/8 h. The germinated seeds grown in transparent boxes were cultured in an incubator with a relative humidity (RH) of 65% and a light intensity of 600 mol m^-2^ s^-1^. Thus, the experiments were designed for four groups, which included a control (neither treated with T6 nor 150 mM NaCl solution), a negative control with 150 mM NaCl stress, a positive control with T6 treatment, and a stressed group T6-NaCl (treated with T6 and 150 mM NaCl). Each treatment was repeated six times.

### Number of Colonies in Wheat Root

Fifteen days after being treated with 150 mM NaCl and the suspension of T6, number of colonies in wheat root was assessed and recorded. The ability of T6 to colonize and grow in association with wheat roots were assessed by determining final T6 densities. Root colonization was assessed following an established protocol (Zhang et al., 2015), where 1 g of surface-sterilized sub-samples of air-dried chopped roots was crushed in 9 ml of sterile water with antibiotics (50 mg^-1^ of streptomycin sulfate) with a sterilized pestle and mortar. The root suspension (10^-3^) was plated onto each of six 9-cm diameter Petri dishes [containing *Trichoderma* medium E (TME); Papavizas and Lumsden, 1982] and incubated in the growth incubator at 25°C for 72 h. The number of T6 density per g of air-dried roots was counted from clear CFU forming on medium after dilution plating of the root suspension. Each treatment was repeated six times.

### Growth Parameters

Wheat seedlings were harvested 15 days after NaCl treatment. Shoots and roots of wheat seedlings were separated and washed with distilled water three times, and then dried and weighed. Root length and weight were determined immediately after being grown for 15 days. For the determination of dry weight, all the samples of wheat seedling shoots and roots were oven-dried at 105°C for 30 min, and then kept at 80°C to obtain a constant weight and were then weighed. Each treatment and control was repeated six times. Relative water content (RWC) of the shoots and roots were recorded by the method of Tian et al. (2015).

RWC(%)=(FW−DW)/FW×100

Where RWC represents relative water content, FW represents fresh weight, and DW represents dry weight.

### Chlorophyll and Proline Content

Chlorophyll was extracted with 80% (v/v) cold acetone from all leaf segments (200 mg), which were frozen at -20°C after 15 days of 150 mM NaCl treatment. The content of chlorophyll a, chlorophyll b, and total chlorophyll in wheat seedling leaves were determined spectrophotometrically according to the method of Lichtenthaler (1987).

Proline content of leaves was determined following the procedure of Bates et al. (1973). After 15 days of NaCl treatment, 0.5 g of fresh wheat seedling leaf samples were homogenized with 10 ml of 3% ASA. After that, 2 ml of AN and 2 ml of GAA were added to 2 ml of the extract and mixed for 1 h at 100°C. The reaction was then stopped by using an ice bath. The reaction mixture was extracted with 4 ml toluene. The absorbance of fraction with toluene aspired from liquid phase was measured at 520 nm. Proline concentration was determined by following a calibration curve and expressed as micromoles proline per gram of fresh weight. Each treatment was repeated for six times.

### Soluble Sugar and Protein Content

All the collected leaf samples from the treatment and control were washed with distilled water three times and cut into small pieces to determine the content of soluble sugar and protein in wheat seedling leaves. Thereafter, the small pieces of wheat seedling leaves were dried, weighed, and placed separately in glass vials which contained 10 ml of 80% (v/v) ethanol, and then placed in a water bath heated at 60°C for 30 min. The filtered extracts were diluted with 80% (v/v) of ethanol to get a total volume of 20 ml. Soluble sugar concentration in the extract was determined by comparison with a standard curve using the criterion of glucose, as described by Giannakoula et al. (2008). Soluble protein content was carried out according to the method described by Bradford (1976). The coomassie brilliant blue G-250 reagent with BSA was regarded as a standard to determine the content of soluble protein in wheat seedling leaves.

### Root Activity and Lipid Peroxidation Degree

Root activity was determined by TTC, as described by method with some modifications (Zhang et al., 2014a). Wheat seedling roots were washed, and then excised at 2 cm in length from the root tips 15 days after NaCl application. Root tips were dried with filter paper and homogenized with liquid nitrogen in an ice cold mortar and pestle. The reaction mixture consisted of 0.5 g samples of root tips, 5 ml of PBS (pH 7.0), and 5 ml of 0.4% TTC in a beaker, with root tips fully immersed in the solution for 1 h at 37°C, then immediately mixed with 2 ml of 1 M sulfuric acid to stop the reaction. The red extraction was moved into a tube making the total volume 10 ml using ethyl acetate. Thereafter, the extraction was added and vortexed for 30 s and centrifuged (1, 000 rpm, 5 min). The extraction was measured at 485 nm against a blank of ethyl acetate. The root activity of wheat seedlings was determined by measuring the activity of dehydrogenase, which present the function to reduce the chemical TTC. The analysis was repeated six times.

The concentration of MDA, a product of lipid peroxidation, was assessed by the method of TBA. The contents of MDA were determined following the method of Madhava Rao and Sresty (2000). Samples of fresh leaves (0.5 g) were homogenized in 10 ml of 0.1% (w/v) TCA. Then, 4 ml of 0.5% (w/v) TBA containing 20% (w/v) TCA was added to 1 ml aliquot of supernatant. The absorbance of the supernatant was recorded at 532 and 600 nm and MDA content was expressed as 1 μmol MDA g^-1^ FW. The content of TBARS was determined as described by Hodges et al. (1999). The concentration of TBARS was calculated based on the absorbance at 532 and 600 nm. All the treatments and control were repeated six times.

### Antioxidant Enzymes Activities

Antioxidant enzymes were extracted at 4°C using 0.5 g tissue from the fresh samples of wheat seedling leaves after 15 days of NaCl treatment. Fresh samples were homogenized with 5 ml of extraction buffer, which contained 0.2 mM EDTA, 0.1 M phosphate buffer (pH 7.8) and 2% polyvinylpyrrolidone. Extracts were centrifuged at 10, 000 rpm for 15 min, and the supernatants were used for determining the activities of antioxidant enzymes. All the analysis was repeated six times.

Superoxide dismutase activity was measured as described by the method of Costa et al. (2002). One unit of SOD activity was defined as the amount of crude enzyme extract that inhibits the reduction of β-nitro blue tretrazolium chloride by the rate of 50% at 560 nm in the spectrophotometer.

Peroxidase activity was assayed by determining the increase of absorbance at 470 nm with guaiacol as the substrate (Kochba et al., 1977). The concentration of protein in the extracts was calculated and carried out as the method described by Lowry et al. (1951).

Catalase activity was assayed according to the method of Cakmak and Horst (1991) with some modifications. The activity of CAT was determined by calculating the decline decomposition of H_2_O_2_ in absorbance at 240 nm.

### Extraction of Total RNA and Analysis of Gene Expression by Quantitative Real Time Reverse Transcriptase-PCR (qRT-PCR)

Total RNA was extracted from different treatment of the wheat seedlings leaves (0.2 g) by using PureLink^®^RNA Mini Kit (Tiangen Biotechnology, Beijing, China). The quality of total RNA was quantified by the UV spectrophotometer. First-strand cDNA was synthesized by Revert Aid TM First Strand cDNA Synthesis Kit (Tiangen Biotechnology, Beijing, China). The qRT-PCR was performed in a 20 μl reaction volume tube using the SYBR ExScript qRT-PCR Kit (Takara, Dalian, China) according to the method described previously by Li et al. (2013) and Qiu et al. (2013). Specific primers for SOD, POD, and CAT genes, and the internal control tubulin gene were used to amplify amplicons specific for wheat seedlings. Specific primers were designed according to wheat EST sequences of candidate proteins available in NCBI (Qiu et al., 2014; Zou et al., 2015), and the DNA sequences of specific primers are provided in **Table 1**. Melting curve analysis of amplification products was performed at the end of each PCR to confirm that only one PCR product was amplified and detected. Gene expression was counted and expressed relative to the expression levels of an internal reference gene actin in each sample using the 2^-ΔΔCt^ method (Livak and Schmittgen, 2001).

**Table 1:** DNA sequences of qRT-PCR primers for determining the antioxidant gene expressed in wheat seedlings under salt stress.

Gene names	Accession number	Premiers sequence (5′–3′)
Actin	AB181991	Forward: CTCTGACAATTTCCCGCTCA
		Reverse: ACACGCTTCCTCATGCTATCC
SOD	JQ613154.1	Forward: CATTGTCGATAGCCAGATTCCTTT
		Reverse: AGTCTTCCACCAGCATTTCCAGTA
POD	X53675.1	Forward: CAGCCCTGTAGCCAACATAAA
		Reverse: GCACTTCCACGACTGCTTTG
CAT	GU984379.1	Forward: TTTGATGGGAGTCTTGTGCTTGTG
		Reverse: ACGGTGAGGGAGTTGTCGTTGTT

### Statistical Analysis

The data was subject to one-way ANOVA using the SPSS package (SPSS V16.0, SPSS, Inc., Chicago, IL, USA). Treatment effects were determined using Duncan’s multiple range test and the significances were expressed at *P* < 0.05.

## Results

### Plant Growth and Relative Water Content

**FIGURE 1:**
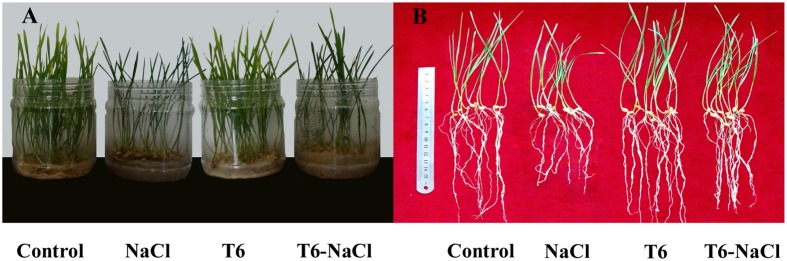
**Effect of salt stress and *Trichoderma longibrachiatum* T6 treatments on (A) wheat seedling growth and (B) wheat root length**. The treatment names are detailed in the footnote of **Table 2**.

Fifteen days after NaCl treatment, the wheat seedling height (**Figure 1A**) and root length (**Figure 1B**) were significantly reduced compared to the control, while the application of the plant-growth-promoting fungi T6 significantly increased wheat seedling height and root length, compared to the NaCl stress treatment. Compared to the control, T6 promoted wheat seedling growth after 15 days without NaCl treatment.

The plant-growth-promoting fungi T6 showed a great ability to colonize the roots of wheat seedlings. Compared to the control and NaCl treatments alone, colonies of T6 were re-isolated from the wheat roots, regardless of whether or not the wheat seeds were soaked with the suspension of T6 under salt stress (**Table 2**). In contrast, there were no colonies re-isolated from the roots in the control or NaCl treatments alone. These observations indicated that the strain of T6 had the ability to colonize the roots of wheat seedlings under salt stress.

**Table 2:** Effects of *Trichoderma longibrachiatum* T6 on number of colonies in wheat roots and plant growth traits under salt stress.

Treatments	Colony densities [CFU g^-1^ of root (×10^5^)]	Plant growth parameters	
		Plant height (cm plant^-1^)	Root length (cm plant^-1^)
Control	0.00 ± 0.00 c	13.30 ± 0.50 b	12.14 ± 0.78 bc
NaCl	0.00 ± 0.00 c	11.03 ± 0.58 c	10.18 ± 0.59 c
T6	5.40 ± 0.47 a	15.23 ± 0.59 a	14.34 ± 0.49 a
T6-NaCl	4.46 ± 0.36 b	12.70 ± 0.61 bc	13.62 ± 0.64 ab

*Mean ± standard error of replicates in a column followed by different letters are significantly different based on Duncan’s multiple range test at *P* < 0.05 (*n* = 12). Control represents wheat seedlings grown under normal condition; NaCl represents wheat seedlings stressed under 150 mM NaCl; T6 represents wheat seeds pretreated with T6 for 12 h before planting without NaCl treatment; T6-NaCl represents wheat seeds pretreated with T6 for 12 h before planting and then stressed under 150 mM of NaCl*.

The concentration of 150 mM of NaCl stress decreased wheat seedling growth. The shoot height and root length decreased by 17 and 16%, respectively, compare to the control (**Table 2**). The shoot and root fresh weights decreased by 33 and 26%, and dry weights decreased by 15 and 19%, respectively, compared to the control (**Table 3**). However, compared to NaCl-stressed plants, the shoot height and root length increased by 15 and 34%, respectively, in plants after being treated with T6 under salt stress (**Table 2**). Also, the shoot and root fresh weights increased by 23 and 22%, and dry weights increased by 10 and 24%, respectively (**Table 3**).

**Table 3:** Effects of *T. longibrachiatum* T6 on wheat seedling weight and relative water content under salt stress.

Treatments	Wheat shoot			Wheat root		
	Fresh weight (g plant^-1^)	Dry weight (g plant^-1^)	Relative water content (%)	Fresh weight (g plant^-1^)	Dry weight (g plant^-1^)	Relative water content (%)
Control	0.271 ± 0.02 b	0.048 ± 0.003 ab	82.16 ± 0.53 a	0.121 ± 0.006 b	0.021 ± 0.001 b	82.61 ± 0.67 ab
NaCl	0.182 ± 0.01 d	0.041 ± 0.001 b	77.75 ± 0.76 c	0.089 ± 0.003 c	0.017 ± 0.002 c	80.65 ± 0.47 b
T6	0.299 ± 0.02 a	0.054 ± 0.004 a	82.00 ± 0.47 a	0.136 ± 0.004 a	0.023 ± 0.002 a	83.01 ± 0.59 a
T6-NaCl	0.224 ± 0.01 c	0.045 ± 0.002 ab	80.08 ± 0.30 b	0.109 ± 0.006 b	0.021 ± 0.001 b	81.27 ± 0.64 ab

*Mean ± standard error of replicates in a column followed by different letters are significantly different based on Duncan’s multiple range test at *P* < 0.05 (*n* = 12). The treatment names are detailed in the footnote of **Table 2***.

Compared to the control, the RWC of shoots and roots were lower for the plants treated with 150 mM of NaCl stress alone, but inversely, the application of T6 significantly increased the water content in wheat shoots and roots (**Table 3**).

### Chlorophyll and Proline Content

Chlorophyll a, chlorophyll b, and total chlorophyll contents were decreased by 15, 17, and 15%, respectively, when treated with 150 mM of NaCl, compared to the control. However, the leaf chlorophyll a, b, and total chlorophyll contents in NaCl-stressed wheat seedlings were reversed to a similar level as the control, after being treated with T6. Leaf chlorophyll contents were lower in the plants under salt stress without T6. Also, compared to the control, the values of chlorophyll a, b, and total chlorophyll contents were increased significantly with the application of T6 without salt stress (**Table 4**).

**Table 4:** Effects of *T. longibrachiatum* T6 on the contents of chlorophyll, proline, soluble sugar, and protein in wheat seedlings under salt stress.

Treatments	Chlorophyll a content (mg g^-1^)	Chlorophyll b content (mg g^-1^)	Total chlorophyll content (mg g^-1^)
**Effect on chlorophyll**			
Control	1.44 ± 0.09 b	0.48 ± 0.06 c	1.92 ± 0.15 b
NaCl	1.23 ± 0.13 c	0.40 ± 0.11 c	1.63 ± 0.11 c
T6	1.59 ± 0.12 a	0.56 ± 0.08 a	2.16 ± 0.20 a
T6-NaCl	1.42 ± 0.16 b	0.54 ± 0.10 b	1.96 ± 0.21 b

**Treatments**	**Proline (μmol g**^-^**^1^ FW)**	**Soluble sugar (mg g**^-^**^1^)**	**Soluble protein (mg g**^-^**^1^)**

**Effect on proline, soluble sugar, and protein**			
Control	15.23 ± 0.29 c	20.58 ± 0.42 c	15.58 ± 0.41 b
NaCl	20.40 ± 0.44 b	17.44 ± 0.35 d	12.74 ± 0.64 c
T6	22.17 ± 0.93 b	24.54 ± 0.67 a	18.56 ± 0.62 a
T6-NaCl	27.63 ± 1.05 a	22.85 ± 0.40 b	17.25 ± 0.84 ab

*Data are mean ± standard error of replicates in a column followed by different letters are significantly different at *P* < 0.05 (*n* = 12), based on Duncan’s multiple range test using one-way ANOVA. The treatment names are detailed in the footnote of **Table 2***.

Compared to the control, the proline content was significantly increased in wheat seedling leaves, after being treated with 150 mM of NaCl solution or with the T6 strain. The highest increase of proline was presented in wheat plants pretreated with T6 under 150 mM NaCl stress, which increased by 35% in leaves with NaCl treatment for 15 days, compared to NaCl-stressed plants (**Table 4**).

### Soluble Sugar and Protein Content

Application of T6 increased the soluble sugar and protein contents in the wheat seedlings grown under salt stress or non-saline stress, compared to the control. However, the content of soluble sugar and protein in wheat seedlings significantly decreased after the treatment of NaCl alone (**Table 4**). In the wheat leaves, the content of soluble sugar and protein decreased by 15 and 18% after the NaCl treatment alone compared to the control, but they increased by 41 and 46% when treated with T6 alone, and increased by 31 and 35%, respectively, when treated with T6 in combination with 150 mM of NaCl, compare to the NaCl-stressed plants. These results showed the application of T6 increased the contents of soluble sugar and protein in wheat seedlings, with the effect on the soluble protein content being greater than the effect on soluble sugar content (**Table 4**).

### Root Activity and Lipid Peroxidation Degree Detection

The root activity of wheat seedlings significantly decreased under salt stress. The root activity decreased by 20% in wheat seedlings treated with 150 mM of NaCl, while the root activity significantly increased after the seedlings were treated with T6 or T6 plus 150 mM of NaCl. T6 had a high effect on the root activity whether the wheat seedlings were under salt stress or non-saline stress (**Table 5**).

**Table 5:** Effect of *T. longibrachiatum* T6 on the root activity and MDA content of wheat seedlings under salt stress.

Treatments	Root activity (μg g^-1^ h^-1^)	MDA content (μmol g^-1^ FW)
Control	225.44 ± 6.45 b	16.79 ± 0.31 c
NaCl	179.56 ± 8.71 c	25.12 ± 1.12 a
T6	259.84 ± 8.11 a	13.87 ± 1.15 c
T6-NaCl	242.46 ± 10.43 ab	21.37 ± 0.52 b

*Data are mean ± standard error of replicates in a column followed by different letters are significantly different at *P* < 0.05 (*n* = 12), based on Duncan’s multiple range test using one-way ANOVA. The treatment names are detailed in the footnote of **Table 2***.

Malondialdehyde, a product of lipid peroxidation, is generally regarded as an indicator of free radical damage to cell membranes caused by oxidative stress. Wheat seedlings inoculated with 150 mM of NaCl and without T6 increased the MDA content in leaves by 50%, compared to the control. However, the seedlings inoculated with the strain of T6 had high efficiency in decreasing the content of MDA under salt stress, compared to NaCl-stressed plants. Thus, the content of MDA decreased by 45 and 15% in T6 alone and T6 plus NaCl treated plants, respectively, compared to NaCl-stressed plants (**Table 5**).

### Antioxidant Enzymes Detection

**FIGURE 2:**
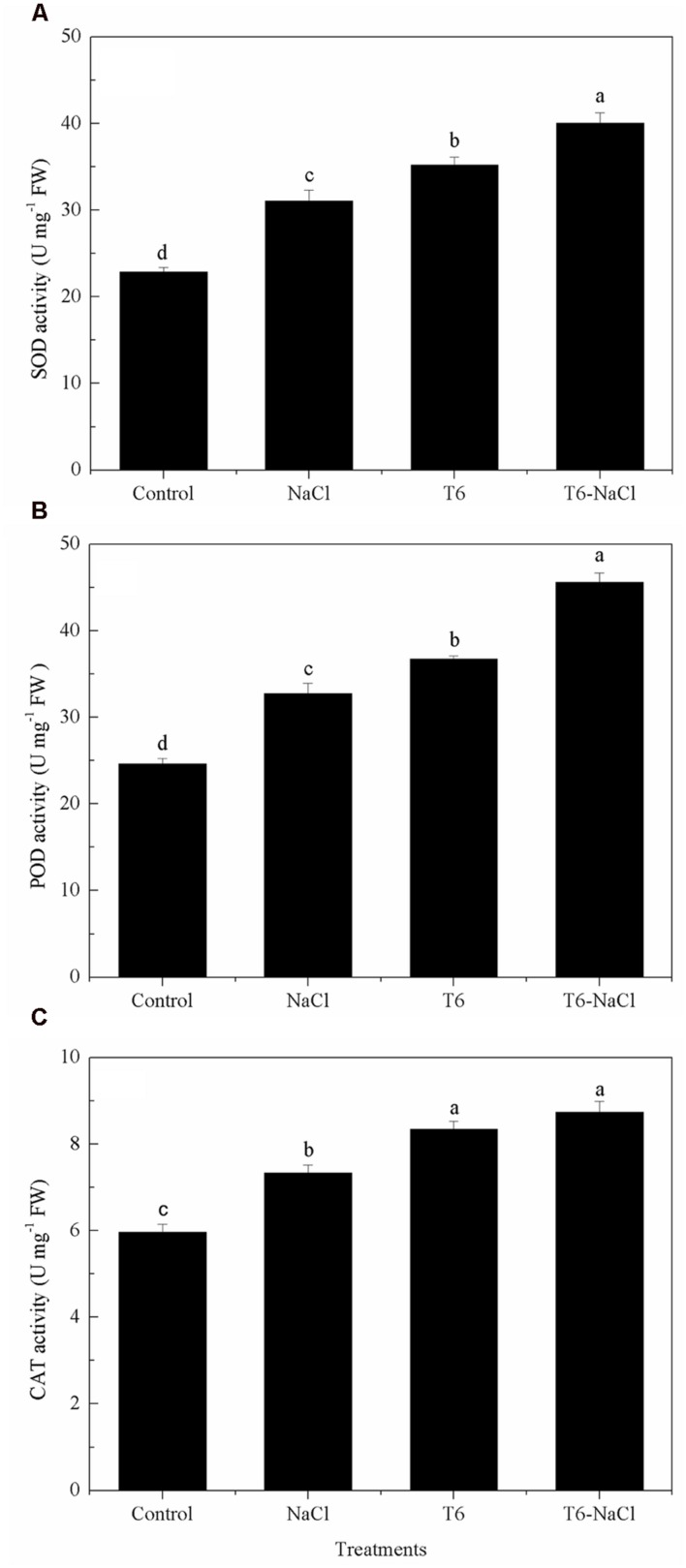
**Effect of *T. longibrachiatum* T6 treatment on the activity of (A) SOD, (B) POD, and (C) CAT in the leaves of wheat seedlings under salt stress**. Small bars represent the standard errors of the means (*n* = 12). Different lowercase letters indicate significant differences at *P* < 0.05 in Duncan’s multiple range test using one-way ANOVA. The treatment names are detailed in the footnote of **Table 2**.

Salt stress significantly induced and increased the antioxidant enzymes activities in wheat seedlings, including the activities of SOD, POD, and CAT (**Figure 2**). Also, the activities of SOD, POD, and CAT were significantly increased after being treated with T6 under salt stress or non-saline stress, compared to the control and the NaCl-stressed plants. Moreover, the application of T6 significantly increased the activities of SOD (**Figure 2A**), POD (**Figure 2B**), and CAT (**Figure 2C**) either in the control group or in the NaCl-stressed wheat seedling leaves; in the control, these values increased by 13, 12, and 14%, respectively; in the NaCl-stressed plants, these values increased by 29, 39, and 19%, respectively, compared to the NaCl treatment alone. The activities of SOD, POD, and CAT were greater with the addition of T6 in the NaCl-stressed plants, compared to the control plants with T6. The activity of CAT in seedlings treated with T6 alone did not differ from seedlings treated with T6 plus NaCl stress treatment, whereas the activities of SOD and POD differed significantly between the two treatments.

### The Level of SOD, POD, and CAT Gene Expression

**FIGURE 3:**
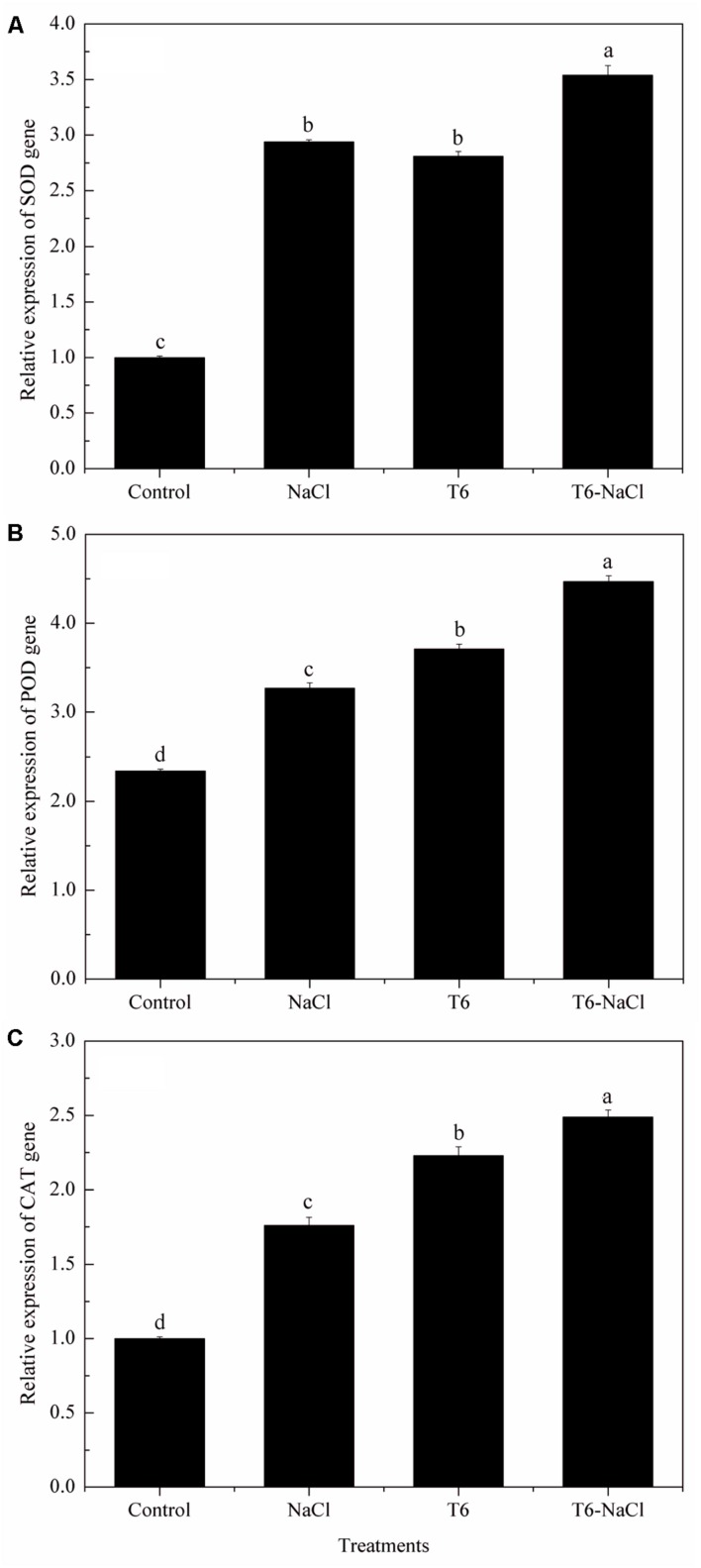
**Effect of *T. longibrachiatum* T6 on the genes of (A) SOD, (B) POD, and (C) CAT expression in the leaves of wheat seedlings under salt stress**. Small bars represent the standard errors of the means (*n* = 12). Different lowercase letters indicate significant differences at *P* < 0.05 in Duncan’s multiple range test using one-way ANOVA. The treatment names are detailed in the footnote of **Table 2**.

Compared to the control plants, there were higher levels of SOD, POD, and CAT gene expression in wheat seedlings after being induced by NaCl stress (**Figure 3**). With the T6 treatment, the gene expression of the SOD (**Figure 3A**), POD (**Figure 3B**), and CAT (**Figure 3C**) were significantly up-regulated whether or not the wheat seedlings were treated with NaCl stress, compared to the control. In contrast, there were no significant differences in the expression levels of the SOD genes in the treatment of the NaCl stress alone or T6 alone (**Figure 3A**).

## Discussion

Previous studies have demonstrated that a high level of salinity is one of the major environmental stress factors that causes biochemical alterations in plants, limits plant growth, and decreases plant productivity (Allakhverdiev et al., 2000; Mahmood et al., 2012). *Trichoderma* species are one of the most versatile opportunistic plant symbionts which can colonize plant roots (Brotman et al., 2013). These symbionts are well-known for their remarkable interactions with host plants and their ability to induce broad-spectrum resistance to plant pathogens (Naseby et al., 2000; Yedidia et al., 2003; Harman et al., 2004). Although, the plant-growth-promoting capability of *Trichoderma* spp. has been previously reported, there is little information concerning plants’ systemic responses induced by T6 under salt stress conditions. Our results demonstrated that the NaCl treatment significantly inhibited wheat seedling growth and development after 15 days and the effect was alleviated substantially with the application of T6. To the best of our knowledge, the present study is the first to discover the role of plant-growth-promoting fungi T6 in enhancing the tolerance of wheat seedlings to salt stress. Also, our study determined the possible mechanism of how plant-growth-promoting fungi T6 alleviated the negative effect of NaCl stress in wheat seedlings. The use of T6 enhanced the tolerance of wheat seedlings to salt stress at physiological and molecular levels.

Shoresh et al. (2010) reported that host roots colonized by *Trichoderma* strains enhanced whole-plant tolerance to biotic and abiotic stresses. The enhancement was indicated by increased plants root growth and nutritional status (Harman et al., 2000), and induced systemic resistance to diseases (Harman et al., 2004). In the present study, we found that T6 has a great ability to colonize the roots of wheat seedlings under salt stress, which significantly improved wheat seedlings growth and development under salt stress. Bae et al. (2009) showed that cacao (*Theobroma cacao*) seedlings which were colonized by *Trichoderma hamatum* isolate DIS 219b enhanced seedling growth and development. In a similar study, Adams et al. (2007) found that the plant saplings grown with *T. afroharzianum* T22 produced more biomass than non-inoculated controls in metal contaminated soil. Our findings are supported by a number of previous observations where *Trichoderma* spp. has the ability to colonize plant roots, establish symbiotic relationships with a wide range of host plants, and promote plant growth and development (Shoresh et al., 2010; Harman, 2011). Moreover, similar results were reported that *Trichoderma parareesei* increased the tomato lateral root development and growth promotion under salt stress conditions (Rubio et al., 2014).

Chlorophyll content is widely used as an indicator of abiotic tolerance in plants. Singh and Gautam (2013) reported that plants exposed to salinity stressful environments decreased chlorophyll concentration, leading to overall growth retardation. In the present study, we found that NaCl-induced stress significantly decreased chlorophyll content in wheat seedlings, but chlorophyll content was reversed back to a similar level as the control in wheat seedlings treated with T6. Similar results were reported in NaCl-stressed soybean (*Glycine max*) and cotton (*Gossypium* spp.) seedlings by other researchers (Simaei et al., 2011; Liu et al., 2014). Tariq et al. (2011) found the oxidation of chlorophyll and chloroplast pigments, as well as the instability of the pigment protein complex in salt stress conditions were the possible reasons for the decrease of chlorophyll content in salinity-stressed wheat seedling leaves. However, we discovered that the content of chlorophyll significantly increased in wheat seedlings with the application of T6 whether or not the seedlings were subjected to salt stress. The latter application may inhibit the production and accumulation of ROS in plant tissue. Similar results were reported in NaCl-treated cucumber (*Cucumis sativus*) seedlings (Qi et al., 2012).

Plant roots are critical for plant growth and development which is attributed to their function and importance in absorption of nutrients and water from soil (Qi et al., 2012). We found NaCl stress had a high effect on the wheat root growth and development, and that salt treatment significantly decreased the root activity. However, the root activity was significantly increased when the plant-growth-promoting fungi T6 was applied to the wheat seedlings either under salt stress or non-saline conditions. A similar phenomenon was found in our previous studies where the strain of *T. longibrachiatum* significantly increased the root activity after wheat seedlings were infected with *H. avenae*, a plant parasitic nematode (Zhang et al., 2014a).

In the present study, the content of proline was increased in wheat seedling grown with NaCl alone, compared to the control. The results from the previous research indicated that the increased level of proline in plants under salt stress condition may have been due to the activation of proline biosynthesis which enhances protein turnover (Khan et al., 2010). Proline is an important nitrogen source that is available for plant recovery from environmental stress and restoration of growth (Trotel et al., 1996), and it can act as an osmolyte that reduces the osmotic potential of the cell and the uptake of toxic ions (Woodward and Bennett, 2005). Thus, proline plays a predominant role in protecting plants from osmotic stress (Khan et al., 2010). An added value from the present study is that the use of the plant-growth-promoting fungi T6 can significantly increase the proline content in wheat seedlings under salt or non-saline stress. Alleviating effects of oligochitosan on salt stress-induced oxidative damage in wheat leaves might be related to its regulation roles in proline levels. In addition, the results from our study indicated that the content of MDA significantly increased in NaCl-stressed wheat seedlings in comparison to the control plants, which is consistent with the findings of Seckin et al. (2008) in wheat. Thereafter, the content of MDA significantly decreased after wheat seeds were soaked in the suspension of T6 before NaCl stress. Taken together, our results are consistent with data from Rawat et al. (2011), who demonstrated that wheat seed biopriming with salinity-tolerant isolates of *T. harzianum* Th-14, Th-19, and Th-13 reduced the accumulation of MDA content, whereas, it increased the proline content in wheat seedlings under both salt and non-saline conditions.

It is widely accepted that osmosis molecules, including soluble sugars and proteins, are important indicators in response to abiotic stress (Azevedo-Neto et al., 2006). The increased accumulation of glucose and sucrose in plants usually indicates a highly protective mechanism against oxidative damage caused by high salinity in the plant environment (Murakeozy et al., 2003; Bartels and Sunkar, 2005). However, most previous studies determined the physiologic role of soluble sugars and utilization by plants. We found that T6 had a highly significant effect on the content of soluble sugar and protein in wheat seedlings either under salt stress or non-saline conditions.

In plants, the overproduction of ROS is considered a biochemical change under salt stress (Younis et al., 2010), which is the most important factor responsible for NaCl-induced damage to macromolecules and plants cellular structures (Özdemir et al., 2004). To alleviate the damage associated with the overproduction of ROS, plants have naturally developed a wide range of enzymatic defense mechanisms to detoxify free radicals and thereby help protect themselves from destructive oxidative damage (Parida et al., 2004; Li et al., 2011). One of the important protective mechanisms in plants is the enzymatic antioxidant system, which involves the simultaneous action of a number of enzymes including SOD, POD, and CAT (Ma et al., 2012). The findings from the present study demonstrate that NaCl stress induced plants produce a higher level of SOD, POD, and CAT activity in wheat seedlings than the control samples. However, the use of T6 increased SOD, POD, and CAT activity in wheat seedlings regardless of salt concentration, which was in accordance with the findings of Ahmad et al. (2015), who demonstrated that the role of *T. harzianum* in Indian mustard (*Brassica juncea*) was to mitigate NaCl stress by an antioxidative defense system. Our results suggested that the coordination of POD and CAT activity along with SOD activity played a central protective role in the O_2_^-^ and H_2_O_2_ scavenging process in wheat seedlings treated with T6. Also, we suggested that the strain of T6 had better O_2_^-^ and H_2_O_2_ scavenging ability than the control in protecting the plants from oxidative damage.

Some plant-beneficial fungi *Trichoderma* species can induce profound impacts or changes in different species of plant gene expression under biotic and abiotic stresses (Mastouri et al., 2010). Increased SOD activity in stressed plants may be attributed to the significantly increased level of ROS, which causes an increase of gene expression responsible for encoding the activity of SOD (Bowler et al., 1992; Shekhawat et al., 2010). Therefore, in the present study, we firstly determined the effect T6 might have had on the expression of antioxidant enzyme genes (SOD, POD, and CAT). We found that the change in expression for the genes of SOD, POD, and CAT had resulted in increased transcription levels which were in accordance with the increase of SOD, POD, and CAT activity; these increases coincided with the trend of the activities of the corresponding enzymes. A number of previous studies have also demonstrated that there is a higher level of gene family expression for genes involved in plant protection against abiotic stresses (Bailey et al., 2006; Alfano et al., 2007) in *Trichoderma* spp. pretreated plants. However, there are no in-depth studies on specific gene expression of wheat seedlings treated with plant-growth-promoting fungi T6. Meanwhile, we have confirmed that ROS generation under salt stress is followed by increased transcription and activity of ROS-scavenging enzymes in T6-challenged wheat plants, which indicated that the important role of ROS was detoxifying cellular survival and regulating plant acclimation (Miller et al., 2010). In addition, ROS also served as a critical signaling molecule in cell proliferation and survival. Similar results have been reported that salinity is one of the environment factors that can change the normal homeostasis of plant cells, and causes an increased production of ROS within plants. The ROS molecule functions as a toxic by-product of stress metabolism and is important in signaling transduction molecules in response to salt stress (Miller et al., 2010).

## Conclusion

In summary, our results indicated that the strain of plant-growth-promoting fungi T6 has a remarkable effect on alleviating the adverse effects of salt stress on wheat seedling growth and development. Multiple tests employed in the study allowed us to explore the possible mechanisms at a physiological and molecular level in which T6 provides the ability of alleviating the suppression effect of salt stress. The mechanisms may include (i) T6 increasing the activity of antioxidative defense system in wheat seedlings to resist salt stress, and (ii) enhancing the relative levels of antioxidant gene expression in the stressed plants. However, there are some issues that need to be addressed in future studies, such as the efficacy of the strain of plant-growth-promoting fungi T6 interactions with other plant species and other abiotic stresses. More detailed studies may be necessary to determine which compound plays the signaling role in T6 that induces systemic changes in the expression of encoding antioxidant enzymes and genes.

## Author Contributions

SZ and YG conceived and designed the experiments with the help of BX. SZ performed most of the salt treatment experiments and prepared the wheat RNA samples. YG and SZ performed qRT-PCR and analyzed the data, with the help of BX. SZ and YG wrote the manuscript. SZ, YG, and BX revised and approved the final manuscript.

## Conflict of Interest Statement

The authors declare that the research was conducted in the absence of any commercial or financial relationships that could be construed as a potential conflict of interest.

## ABBREVIATIONS

AN: acid ninhydrin
ASA: aqueous sulfosalicylic acid
BSA: bovine serum albumin
CAT: catalase
CFUs: colony-forming units
GAA: glacial acetic acid
H_2_O_2_: hydrogen peroxide
MDA: malondialdehyde
PBS: phosphate buffer solution
POD: peroxidase
ROS: reactive oxygen species
SOD: superoxide dismutase
TBA: thiobarbituric acid
TCA: trichloroacetic acid
TTC: triphenyl tetrazolium chloride
T6: *Trichoderma longibrachiatum* T6
